# Effects of Length of Abstinence on Decision-Making and Craving in Methamphetamine Abusers

**DOI:** 10.1371/journal.pone.0068791

**Published:** 2013-07-24

**Authors:** Guibin Wang, Jie Shi, Na Chen, Lingzhi Xu, Jiali Li, Peng Li, Yan Sun, Lin Lu

**Affiliations:** National Institute on Drug Dependence, Peking University, Beijing, China; University of Granada, Spain

## Abstract

**Rationale:**

The majority of drug abusers are incapable of sustaining abstinence over any length of time. Accumulating evidence has linked intense and involuntary craving, Impulsive decision-making and mood disturbances to risk for relapse. However, little is known about temporal changes of these neuropsychological functions in methamphetamine (METH)-dependent individuals.

**Objectives:**

To investigate the effect of length of abstinence on decision-making, craving (baseline and cue-induced), and emotional state in METH-addicted individuals.

**Methods:**

In this cross-sectional study, 183 adult METH-dependent patients at an addiction rehabilitation center who were abstinent for 6 days (n = 37), 14 days (n = 33), 1 month (n = 31), 3 months (n = 30), 6 months (n = 26), or 1 year (n = 30) and 39 healthy subjects were administered the Iowa Gambling Task (IGT) to assess decision-making performance. Depression, anxiety, and impulsivity were also examined. One hundred thirty-nine METH abusers who were abstinent for the aforementioned times then underwent a cue session, and subjective and physiological measures were assessed.

**Results:**

METH dependent individuals who were abstinent for longer periods of time exhibited better decision-making than those who were abstinent for shorter periods of time. And self-reported emotional symptoms improved with abstinence. METH abusers’ ratings of craving decreased with the duration of abstinence, while cue-induced craving increased until 3 months of abstinence and decreased at 6 months and 1 year of abstinence.

**Conclusions:**

We present time-dependent alterations in decision-making, emotional state, and the incubation of cue-induced craving in METH-dependent individuals, which might have significant clinical implications for the prevention of relapse.

## Introduction

Methamphetamine (METH) abuse is a worldwide problem that imposes substantial global public health and costly social burdens [1], [2]. Relapse rates following psychosocial and pharmacological treatments are high in METH-addicted individuals and remains the major problem [3], [4], [5]. A better understanding of the mechanisms that increase relapse susceptibility could contribute to the development of more effective relapse prevention treatments in METH dependence.

There is a growing evidence of potential psychological problems and cognitive impairments as a result of prolonged METH use in humans [6]. Individuals with substance dependence frequently show signs of impaired emotion and neurocognitive functions, even after prolonged abstinence from drug use [7], [8], [9]. But few have examined the role of abstinence on neurocognitive deficits, and the results is mixed [10].

### Decision-making

Neuropsychological and personality research indicated that addiction is associated with elevated scores on questionnaires that measure impulsivity and impaired performance on laboratory tasks of impulsivity [11], [12], [13], [14]. The behavioral analysis of impulsivity in humans has predominantly used three paradigms: decision-making, response inhibition, and reflection impulsivity [14]. The Iowa Gambling Task (IGT) was specially designed to measure decision-making impairment in the laboratory, resembling the decisions made in real life in terms of reward, punishment, and the uncertainty of outcomes [15]. An impairment of decision-making has been found in nearly all substance-dependent populations, including people addicted to alcohol [16], cannabis [17], METH [18], cocaine [19], and heroin [13]. Our previous work have shown that the short-term abstinent heroin-dependent patients performed worse on the IGT compared with the long-term abstinence groups [13]. However, whether the length of abstinence affects decision-making ability in METH-addicted individuals is unknown.

### Craving

Theoretical perspectives and clinical survey reports indicated that drug-related cues are a key factor that increases the risk of relapse in substance-dependent subjects [5], [20]. Abstinent heroin-addicted patients who relapsed exhibited greater drug cue-induced reactivity compared with nonrelapsers and controls [21]. Cue-induced drug seeking increases with longer periods of abstinence, termed the “incubation of drug craving” [22], which was used to account for the persistent relapse after prolonged abstinence. The incubation of craving has been reported in rats that were trained to self-administer cocaine [22], heroin [23], METH [24], nicotine [25], and oral sucrose [26] and in human smokers [9]. Whether the incubation of cue-induced craving occurs in METH abusers and how long it remains require further studies.

### Emotional Symptoms

Chronic METH abuse can cause mood disturbances. Symptoms of depression and anxiety are common among METH-dependent and -withdrawn individuals [27], [28] and associated with craving for the drug, which may exacerbate relapse to METH use [29]. Previous studies found that depressive symptoms varied considerably in intensity and duration, which resolved within 2 to 3 weeks [30], [31], [32].

In the present study, we employed cross-sectional and experimental approaches to investigate decision-making performance, emotional state, and the desire for drug confronted with METH-related cues or not in METH-dependent subjects at different periods of abstinence.

## Materials and Methods

The study was approved by the Peking University Research Ethics Board. The participants were given detailed study descriptions, provided written informed consent, and received monetary compensation.

### Subjects

This was a cross-sectional study conducted at Zhongshan Compulsory Addiction Rehabilitation Center, Guangdong province. One hundred eighty-three male inpatients who were formerly addicted to METH and drug-free for 6 days (6 d, n = 37), 14 days (14 d, n = 33), 1 month (1 m, n = 31), 3 months (3 m, n = 30), 6 months (6 m, n = 26) and 1 year (1 y, n = 30) were administered the Iowa Gambling Task (IGT) to assess decision-making performance. And 139 METH abusers who were abstinent for the aforementioned times (6 d: n = 24; 14 d: n = 26; 1 m: n = 19; 3 m: n = 20; 6 m: n = 20; 1 y: n = 29) underwent cue sessions. The last use of METH of drug abusers was on the day before study entry and urine sample for kind of drugs abused verification were provided before admitting. In the center, METH abusers have no chance to find and use METH. So the abstinence period can be strictly controlled. Prior to enrollment, all of the subjects were screened using the Structured Clinical Interview for the *Diagnostic and Statistical Manual of Mental Disorders*, 4th edition (DSM-IV) and had to provide answers to questions regarding their METH abuse, which was based on when they were actively using. All of the subjects had a history of a DSM-IV diagnosis of METH dependence and did not have any current medical illness that required pharmacological treatment or any other substance use disorder with the exception of nicotine dependence. Other exclusion criteria included a history of head injury, severe mental illness, psychosis and neurological disorders. All of the METH participants identified smoking as the primary route of METH administration. Each participant was permitted to be in only one abstinence-period test. Thirty-nine education-matched male healthy controls were recruited through advertisements. All participants are aged between 18 and 40 years.

### Neuropsychological Measures

#### Iowa Gambling Task

Decision-making was assessed by the original version of the IGT. The subjects were asked to choose among four decks of cards and accumulate as much money as possible by picking one card at a time. To win money, the subjects must discover which are the most advantageous decks and preferentially select cards from those decks. Deck A and B are associated with high immediate wins but larger future penalties that results in a net loss over time (i.e., the disadvantageous decks). Deck C and D yields lower immediate wins but smaller future penalties, such that the participants gradually accumulate a profit by choosing these decks (i.e., the advantageous decks). The outcome measure for this task is the net score, which is calculated as the number of cards from the disadvantageous decks subtracted from the advantageous decks. Therefore, a positive score reflects a tendency to make good decisions.

### Cue Sessions

The main design was between-subjects. During the cue sessions, neutral cues or METH cues were presented first randomly. Baseline physiological and subjective measurements were collected prior to the first cue exposure. After baseline measures, participants were exposed to the first cues. Immediately after exposure, cardiovascular (CV) and subjective measurements were collected. There was a 20 min of free time between the two cues sessions. Physiological and subjective measurements were again collected just before participants started the second cue set, which was followed by CV and subjective measurements. Subjects who reported craving after METH cue exposure were debriefed at the end of assessments and remained in the research clinic until their levels of craving returned to baseline levels. For METH cues, the subjects viewed (1) 32 photographs of individuals procuring and using METH, with each presented for 7 s in a slide show, (2) a 5 min video in which METH abusers made drug paraphernalia and used METH, and (3) paraphernalia placed directly on the table in front of the subjects. Neutral cues consisted of 32 neutral pictures without explicit METH cues and a 5 min video of a natural landscape. Subjective measures included a 10-point visual analog scale (VAS), in which the participants marked a score of 1 (not at all) to 10 (extremely high) in response to the question “How much do you feel the urge to use METH?”.

### Questionnaires and Neuropsychological Measures

#### Hamilton Anxiety Scale (HAMA)

This scale consisted of 14 items, each defined by a series of symptoms. Each item is rated on a five-point scale, ranging from 0 (not present) to 4 (severe) [33]. It was cited from Chinese classification and diagnostic criteria of mental disorder-3rd edition (CCMD-3).

#### Beck Depression Inventory–2nd edition (BDI-II)

This scale **comprises** 13 multiple-choice items that assess the presence and severity of depressive symptoms in clinical and nonclinical samples. It was cited from Handbook of Behavioral Medical Scales (2005).

#### Barratt impulsiveness scale

The subjects were administered the Chinese version of the Barratt Impulsiveness Scale, version 11 (BIS-11), a validated 30-item questionnaire that conceptualizes impulsiveness in three main dimensions [34]: attention impulsiveness, motor impulsiveness, and non-planning impulsiveness. The Chinese version of the BIS-11 was shown to have good internal consistency and test-retest reliability by the Medical Psychological Research Centre of the Second Xiangya Hospital, China [13].

#### d2 test of attention/psychomotor speed

From a series of “d” and “p” letters with one or two dashes above and/or below each letter, the participants were asked to mark the “d” accompanied by two dashes as quickly and accurately as possible**.** A summary test score represents the total number of correctly marked “d” letters minus the number of errors [35]. The Germany version of d2 test was used.

#### Working memory

The digit span task derived form the Wechsler Adult Intelligence Scale-III was used to evaluate working memory. The experimenter reads a string of digits aloud and the subject is instructed to immediately recall those digits either in the forward position or in the backward position. The series of digits were incrementally increased by one digit every time the subject performs the task correctly. The task was stopped when **the subjects** failed to recall two trials with the same number of digits. Score was defined as the total number of digits the subject could correctly recall in the exact order they were presented or in reverse order.

### Cardiovascular Measures

Systolic and diastolic blood pressure was measured with an arm cuff connected to a 9062D monitor (Baozhong Biotechnology, Beijing, China). Heart rate was continuously measured with a fingertip pulse sensor connected to an SD-700 monitor.

### Statistical Analysis

All of the data were analyzed using SPSS 17.0 software. One-way analysis of variance (ANOVA) was used for between-group comparisons of demographic characteristics (i.e., age, years of education, duration of METH use, typical dose and frequency of use every month), neuropsychological task performance (i.e., working memory and d2 test of attention), psychiatric symptoms (including anxiety, depression, and impulsivity), baseline craving scores and physiological measures. **IGT performance was analyzed using one-way Analysis of covariance (ANCOVA) models with age and education as covariates**. To assess whether METH cues were effective, we used *t*-tests to compare within-session changes in the **craving scores** (postcue - precue) for METH cues *vs*. neutral cues. Only those METH abusers whose craving ratings increased after the METH-related cures exposure were included in the main analyses. Group differences were assessed using an ANOVA. **We collected subjective measurement and CV as baseline before each type of cues was exposed, and the dependent variables were within-session changes in the craving scores, with METH and neutral cues analyzed separately**. A similar approach tested group differences in baseline cue session craving [9]. Values of *p*<0.05 were considered statistically significant.

## Results

### Effects of Length of Abstinence on Decision-making in METH addicts

#### Demographic and clinical characteristics

The demographic and clinical characteristics of the healthy control and METH-dependent subjects are shown in Table 1. No significant between-group differences were found with regard to age, level of education, drug use history (i.e., duration, dose, and frequency per month), and working memory.

**Table 1:** Demographic characteristics of subjects.

Characteristic	Control(*n* = 39)	6 day(*n* = 37)	14 day(*n* = 33)	1 month (*n* = 31)	3 month (*n* = 30)	6 month (*n* = 26)	1 year (*n* = 30)	*p* value
Age (years)	30.00±1.40	27.11±1.06	26.76±1.04	29.93±1.17	26.06±1.11	30.00±1.16	28.87±1.12	0.06
Education (years)	9.77±0.41	8.71±0.33	9.18±0.41	8.10±0.42	8.62±0.40	8.62±0.45	8.47±0.37	0.065
Duration of METH use (months)	–	18.9±4.20	21.38±2.77	25.93±3.61	20.93±3.84	26.77±4.72	17.87±2.64	0.46
Average dose (g)	–	0.71±0.13	0.47±0.07	0.58±0.09	0.60±0.08	0.63±0.10	0.67±0.11	0.60
Frequency per month	–	18.31±1.59	18.35±1.62	18.47±1.83	18.21±1.93	17.11±2.21	16.87±1.85	0.98

#### Decision-making ability

**Figure 1:**
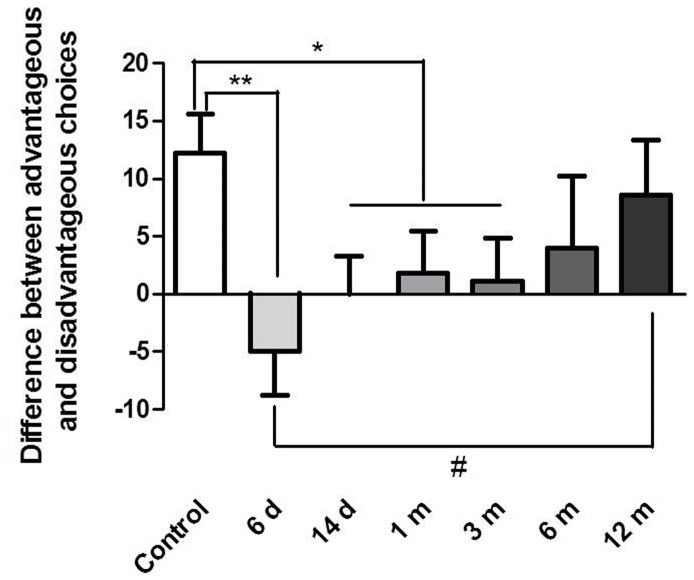
Decision-making ability on the Iowa Gambling Task (IGT) in METH abusers at different abstinence times and healthy controls over 100 card choices. Differences between advantageous and disadvantageous choices ([C+D] - [A+B]) are expressed as mean ± SEM. *p<0.05, differences between METH abusers groups and normal controls (*post hoc t*-test). ^#^ *p*<0.05, differences between 6 d and 12 m abstinence group.

Since the age and education are almost statistically different between the METH and control groups (*p* = 0.06 and *p* = 0.065), the age and education was used as covariate in the statistical analysis. Fig. 1 shows the IGT net scores in former METH dependent individuals and healthy control subjects. One-way **ANCOVA** revealed significant differences in decision-making ability between the seven groups (*F*_6,215_ = 2.356, *p*<0.05). *Post hoc* tests showed that the 6 d and 14 d abstinence groups selected significantly more cards from the disadvantageous decks than healthy control subjects (*p = *0.001, *p = *0.033), while 1 m and 3 m abstinence groups selected almost statistically more disadvantageous cards than healthy subjects (*p = *0.063, *p = *0.081). Moreover, 6 d abstinence group differed from 12 m abstinence in IGT score (*p* = 0.007).

### Effects of Length of Abstinence on Emotional Ratings, Impulsivity, and Neuropsychological Task Performance

#### Anxiety, depression, working memory, and attention

**Figure 2:**
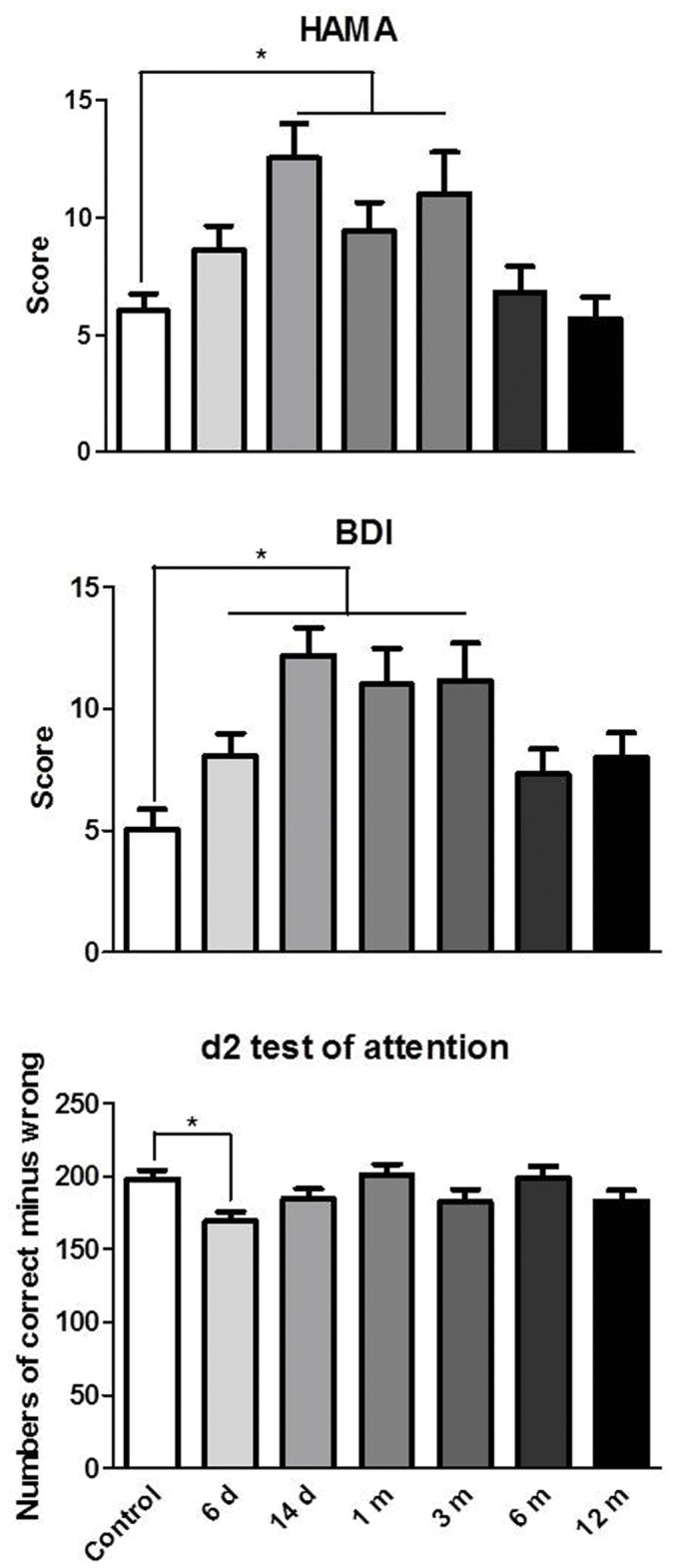
Anxiety, depression, and attention in the subjects. *p<0.05, significant difference between METH abusers and healthy controls.

One-way ANOVA revealed a significant effect of group on anxiety (*F*_6,215_ = 4.53, *p*<0.0005), depression (*F*_6,215_ = 5.12, *p*<0.0001), and attention (*F*_6,215_ = 2.67, *p*<0.05). *Post hoc* tests showed that the 14 d, 1 m, and 3 m groups had more anxiety symptoms, while the 6 d, 14 d, 1 m, and 3 m abstinence groups had less depressive symptoms. And the 6 d abstinence group had an impaired attention level compared with healthy controls (all *p*<0.05). No significant differences were found in working memory (data not shown; Fig. 2).

#### Impulsivity

**Figure 3:**
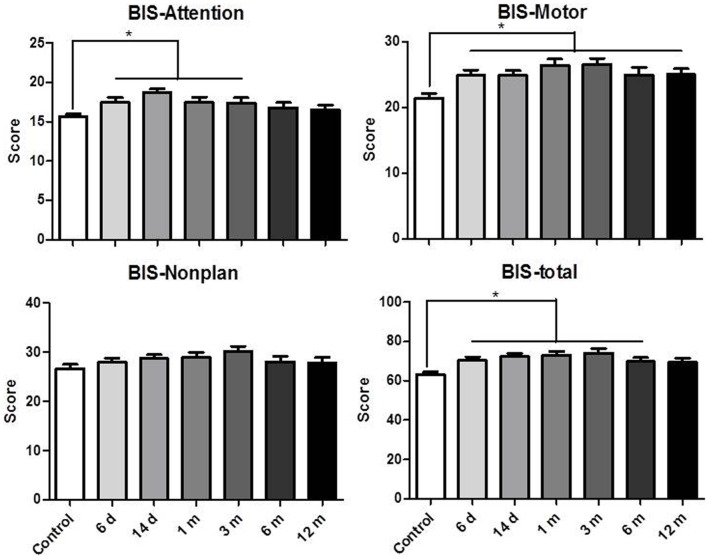
Self-reported impulsivity on the BIS-11 in the subjects. *p<0.05, significant difference between METH abusers and healthy controls.

The BIS-11 scores are presented in Fig. 3. A statistically significant difference was found in self-reported total impulsiveness (*F*_6,215_ = 5.55, *p*<0.0001), motor impulsiveness (*F*_6,215_ = 5.71, *p*<0.0001), and attention impulsiveness (*F*_6,215_ = 3.39, *p*<0.01) between the METH abusers and controls. *Post hoc* tests revealed that the 6 d, 14 d, 1 m, 3 m, and 6 m abstinence groups had higher total scores, while the 6 d, 14 d, 1 m, and 3 m abstinence groups had higher attention scores. And all of the METH groups had higher motor scores than controls.

### Effects of Length of Abstinence on Craving in METH-dependent Subjects

#### Demographic and clinical characteristics

Table 2 presents the demographic and drug use characteristics of the METH addicts enrolled in the cue-exposure experiment. The subjects were well-matched with regard to age, years of education, duration of METH use, average dose and frequency of METH use.

**Table 2:** Demographic characteristics of METH-dependent subjects.

Characteristic	6 day (*n* = 24)	14 day (*n* = 26)	1 month (*n* = 19)	3 month (*n* = 20)	6 month (*n* = 20)	1 year (*n* = 29)	*p* value
Age (years)	28.43±1.41	27.38±1.27	29.95±1.63	26.7±1.38	30.9±1.43	29.67±1.26	0.28
Education (years)	7.65±0.48	9.23±0.50	7.79±0.43	8.40±0.55	7.89±0.70	8.27±0.50	0.29
Duration of METH use (months)	24.83±6.86	27.77±3.84	20.06±4.04	25.30±4.03	22.45±5.54	17.23±2.63	0.55
Average dose (g)	0.65±0.13	0.52±0.08	0.58±0.12	0.53±0.09	0.72±0.24	0.72±0.11	0.81
Frequency per month	19.6±2.23	19.69±1.88	20.53±2.43	16.7±2.06	16.25±2.46	17.80±1.88	0.68

#### Baseline craving

**Figure 4:**
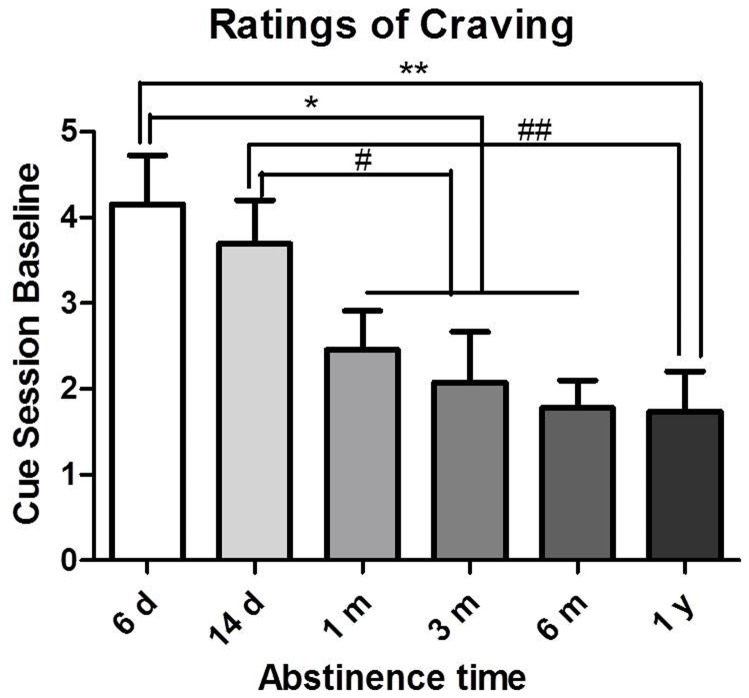
Effects of length of abstinence on baseline (nonprovoked) METH craving. *p<0.05, **p<0.01, compared with 6 d group; ^#^ *p*<0.05, ^##^ *p*<0.01, compared with 14 d group. The data are expressed as group means (± SEM).

Ratings of craving in METH-dependent subjects at different abstinence periods before cue presentations are shown in Fig. 4. The participants’ baseline craving decreased with abstinence (*F*_5,135_ = 4.06, *p*<0.005). *Post hoc* tests revealed that the 1 m, 3 m, 6 m, and 1 y abstinent METH abusers had lower craving than the 6-day group (1 m, 3 m, and 6 m: *p*<0.05; 1 y: *p*<0.01). The 6 m and 1 y groups had lower craving than the 14 d group (6 m: *p*<0.05; 1 y: *p*<0.01).

#### Cue-induced craving

**Figure 5:**
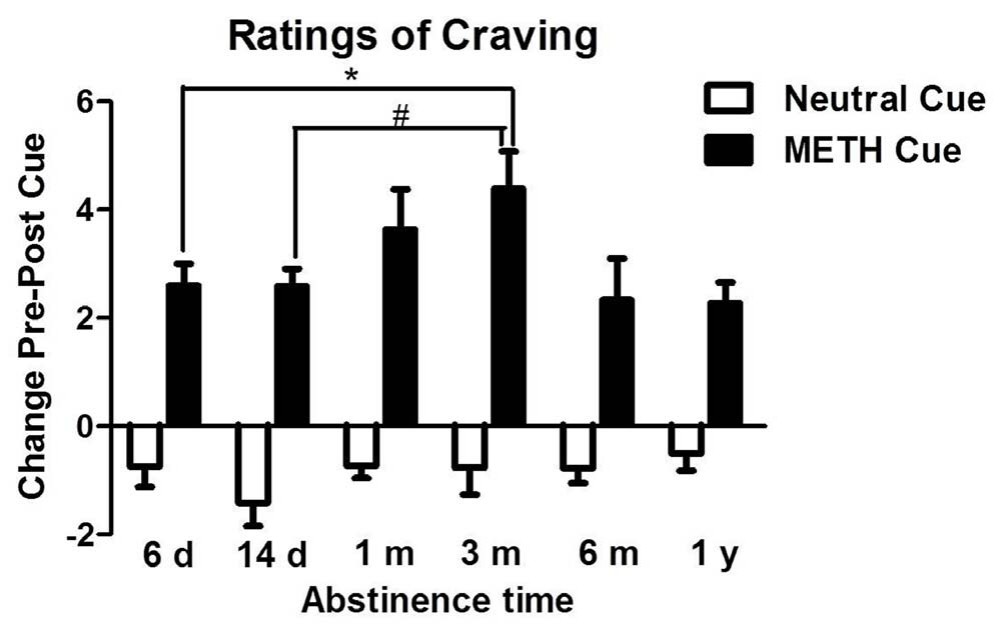
Effects of exposure to METH and neutral cues on self-reported craving as a function of length of abstinence. *p<0.05, compared with 3 m group. The data are expressed as group means (± SEM).

As shown in Fig. 5, the effects of length of abstinence on cue-induced craving in METH-dependent subjects were significantly different (*F*_5,133_ = 2.62, *p*<0.05). Within 3 m of METH abstinence, cues-induced craving increased as the length of abstinence increased. The 3 m group had higher responsivity to the cues than the 6 d and 14 d groups (*p*<0.01), and the responsivity decreased with 6 m and 1 y of abstinence (*p*<0.05).

#### Effects of cues on physiological measures in METH abusers

The effects of length of abstinence on cue-induced physiological measures, including heart rate, systolic blood pressure and diastolic blood pressure, were not significantly different (data not shown).

## Discussion

The present study presented three characteristics of the effects of length of abstinence on METH abusers: impaired impulse control, craving, and emotion. METH dependent individuals who were abstinent for longer periods of time exhibited better decision-making and emotions than those who were abstinent for shorter periods of time. Cue-induced craving increased with increased abstinence up to 3 months.

Some degree of recovery after protracted abstinence has been shown in neuroimaging studies in abstinent METH abusers [36], [37], [38], [39]. However, previous studies on relationship of cognitive function with sustained abstinence from METH use in METH-dependent individuals are inconsistent [10]. Two of three cross-sectional studies of abstinence suggested that some cognitive functions improve with abstinence [38], [40], and a longitudinal study that assessed 1 year of abstinence found METH-dependent individuals who were neuropsychologically impaired at baseline demonstrating significant improvement with abstinence [41]. These suggested that partial recovery may be possible in some cognitive domains, other METH-associated cognitive deficits may be irreversible. Our previous study have shown that stress can exacerbate an already existing impairment of decision-making or unmask a latent one in individuals recovering from heroin dependence [13], which suggested the recovery of decision-making in normal circumstances may not represent the overall recovery of function. No differences were found in working memory among the groups, suggesting that decision-making and other cognitive functions may show differential recovery trajectories. Attention is an important prerequisite for learning, and IGT involves exploratory learning via rewards and penalties. So the impaired attention of METH at 6 days abstinence may exacerbate the impulsive decision-making.

We found that cue-induced craving in METH abusers increased with prolonged abstinence up to 3 months, even as baseline craving declined. This finding is broadly consistent with nonhuman incubation studies that used lever-pressing to measure drug seeking and craving [42] and human smokers [9]. Cue-induced craving decreased at 6 months and 1 year of abstinence, which is also consistent with a rat study in which the incubation phenomenon was long-lasting but not permanent [43]. Recent research found that heroin relapsers exhibited greater drug cue-induced subjective responses compared with both nonrelapsers and control subjects [21]. So the present study suggested that the risk for relapse may increase with an increased length of abstinence up to 3 months. Brain-derived neurotrophic factor and glial cell line-derived neurotrophic factor in the ventral tegmental area, neuronal activation of the ventral medial prefrontal cortex, extracellular signal-regulated kinase activation in the central nucleus of the amygdala, and glutamate receptor 2-lacking 2-amino-3-(5-methyl-3-oxo-1,2- oxazol-4-yl) propionic acid receptors in the nucleus accumbens have been shown to play important roles in the incubation of cocaine craving [42], but the neurobiological mechanisms of METH incubation need further investigation.

Depressive symptoms after the cessation of stimulant abuse have been associated with reduced treatment retention [44]. One study in Filipino male METH users found that negative affect and craving predicted relapse [45], while the antidepressant sertraline delayed relapse in recently abstinent cocaine-dependent patients with depressive symptoms [46]. Diagnosing and monitoring depression in drug abusers and the timely remediation of post-detoxification depression symptoms may help in the prevention of drug relapse. In METH abusers, self-reports of depressive symptoms covaried positively with relative glucose metabolism in limbic regions, and ratings of state anxiety covaried negatively with relative activity in the anterior cingulate cortex and left insula [27], suggesting potential targets for therapeutic intervention. Nakama et al. found that depression and anxiety were significantly correlated with METH craving [29]. Therefore, METH abusers may benefit from treatment of their psychiatric symptoms to minimize craving and subsequent relapse to drug use.

The limitation of the present study should be noted. First, the design did not include a group that was enrolled for a within-subjects component (i.e., IGT performance and cue-induced craving were tested in only one group at different abstinence times). Thus we can not resolve conclusively the pre-existing versus acquired nature of the observed alterations. Second, longitudinal designs would enhance our ability to provide general conclusions regarding the associations between neurocognitive function and abstinence times. However, a previous study found that the incubation of cue-induced craving in smokers was weaker in a within-subjects design than in a between-subjects design, which may be attributable to some extinction of the conditioned response in the laboratory in within-subjects assessments [9].

### Conclusion

In summary, we found that impulsive decision-making, craving, and psychiatric symptoms in METH abusers abated with a longer duration of abstinence, but cue-induced craving did not decrease over an extended period of abstinence and actually increased with 3 months of abstinence. These findings suggest that the risk of relapse in drug abusers does not decline with abstinence, and concurrent interventions that mitigate stress and cue reactivity should be extended to a longer duration.
